# Role of the triad of procalcitonin, C-reactive protein, and white blood cell count in the prediction of anastomotic leak following colorectal resections

**DOI:** 10.1186/s12957-022-02506-4

**Published:** 2022-02-12

**Authors:** Haidi Abd El Zaher, Waleed M. Ghareeb, Ahmed M. Fouad, Khaled Madbouly, Hamada Fathy, Tomas Vedin, Marcus Edelhamre, Sameh H. Emile, Mohammed Faisal

**Affiliations:** 1 Surgical Oncology Unit, Department of Surgery, Faculty of Medicine, Suez Canal University Hospital, Ismailia, Egypt; 2 Gastrointestinal Surgery Unit, Department of Surgery, Faculty of Medicine, Suez Canal University Hospital, Ismailia, Egypt; 3 Faculty of Medicine, Suez Canal University, Ismailia, Egypt; 4 Department of Public Health, Community Medicine, Occupational & Environmental Medicine, Faculty of Medicine, Suez Canal University, Ismailia, Egypt; 5 Colorectal Surgery Unit, Alexandria University, School of Medicine, Alexandria, Egypt; 6 Department of Surgery, Helsingborg Hospital, University of Lund 251 87, Helsingborg, Sweden; 7 Colorectal Surgery Unit, General Surgery Department, Mansoura University Hospital, Mansoura, Egypt; 8 General Surgery Department, Sahlgrenska University Hospital, Gothenburg, Sweden

**Keywords:** Procalcitonin, C-reactive protein, Anastomotic leakage, Colorectal surgery, Biomarker

## Abstract

**Purpose:**

The enhanced recovery after surgery (ERAS) program expedites patient recovery after major surgery. This study aimed to investigate the role of the triad of procalcitonin (PCT), C-reactive protein (CRP), and white blood cells (WBC) trajectories as a predictive biomarker for the anastomotic leak (AL) after colorectal surgery.

**Method:**

Patients who had colorectal anastomosis were prospectively included. Postoperative clinical and laboratory parameters and outcomes were collected and analyzed. The 5-day trajectories of PCT, CRP, and WBC were evaluated. Based on the trajectory of the three biomarkers, we compared patients with and without AL as detected during the first 30 days after surgery using the area under receiver operator characteristic curves (AUC) for logistic estimation.

**Results:**

This study included 205 patients, of whom 56% were men and 43.9% were women with a mean age of 56.4 ± 13.1 years. Twenty-two patients (10.7%) had AL; 77.3% underwent surgery, and 22.7% were treated with drainage and antibiotics. Procalcitonin was the best predictor for AL compared to CRP and WBC at three days postoperatively (AUC: 0.84, 0.76, 0.66, respectively). On day 5, a cutoff value of 4.93 ng/mL for PCT had the highest sensitivity, specificity, and negative predictive value. The predictive power of PCT was substantially improved when combined with either CRP or WBC, or both (AUC: 0.92, 0.92, 0.93, respectively).

**Conclusion:**

The 5-day trajectories of combined CRP, PCT, and WBC had a better predictive power for AL than the isolated daily measurements. Combining the three parameters may be a reliable predictor of early patient discharge, which would be highly beneficial to ERAS programs.

## Introduction

Enhanced recovery after surgery (ERAS) programs incorporates a panel of perioperative protocols and medications. The use of a minimal access approach, pain killers, antiemetic medications, and rehabilitation are commonly used measures in ERAS programs. Overall, the main aim of ERAS is to diminish physiological stress, promote the early return of total capacity, and decrease healthcare costs by shortening the length of hospital stay [1].

The rate of anastomotic failure varies according to the site of anastomosis. The overall incidence of colorectal anastomotic leakage (AL) ranges between 2% and 14% when surgery is performed by an experienced surgeon [2–6]. Early AL usually becomes clinically evident between 5 and 7 days postoperatively [7]. Dehiscence of colorectal anastomosis may increase the local recurrence rate of malignant tumors and postoperative mortality secondary to peritonitis and septicemia [8]. The interest in identifying a biological marker for the early detection of AL is growing [9]. Such a marker could play a vital role in modern fast-track multimodal protocols, allowing safe and early discharge of patients after colorectal surgery with a low readmission rate. C-reactive protein (CRP) has been identified as a valid parameter for detecting postoperative infectious complications after rectal resection [10]. A serum CRP level greater than 12.4 mg/dL on postoperative day (POD) 4 is considered predictive of septic complications [11]. According to a recent analysis, the changes in the trajectory of CRP levels might be more beneficial than a snipped point.

Moreover, the trajectory has negative predictability of up to 99.3% [12]. Another interesting biomarker is procalcitonin (PCT), the prohormone of calcitonin produced by parafollicular C cells in the thyroid. Typically, it has a low plasma concentration in healthy individuals (0.01–0.05 ng/mL), and it increases during severe generalized bacterial, parasitic, or fungal infections, but not in noninfectious inflammatory reactions [13]. Procalcitonin has been described as an early, sensitive, and specific marker of sepsis [14]. Moreover, the plasma concentration of PCT has been used as an early predictor of infection in acute pancreatitis [15], secondary peritonitis, and infectious complications after thoracic, esophageal, and cardiac surgeries [16]. In addition, elevated white blood cell (WBC) count is associated with AL after gastrointestinal surgeries [17, 18]. Therefore, this study was conducted to evaluate the utility of CRP, PCT, and WBC count trajectories, as separate and combined biomarkers for predicting AL after colorectal surgery.

## Patients and methods

### Study design and setting

The present study is a prospective cross-sectional study on consecutive patients who underwent elective or emergency surgery with a colorectal anastomosis between March 2018 and March 2020 at the Surgical Oncology Unit of Suez Canal University Hospitals. All eligible patients provided written informed consent before inclusion in the study. The research ethics committee has approved the current study in the Faculty of Medicine Suez Canal University with reference number (#8037). The study is registered to www.clinicaltrials.gov under number: NCT0515902

### Selection criteria and sample size calculation

We included adult patients of either sex who underwent colorectal surgery entailing an anastomosis. The exclusion criteria included patients younger than 18 years, those with an active infection at the time of surgery, those who had received chemotherapy or radiotherapy, and those on long-term corticosteroid therapy. The sample size was calculated using online software for sample size calculation for observational studies (http://www.raosoft.com/samplesize.html). In light of the population size, referred to as the number of patients having a colorectal anastomosis in our hospital per year (400–420 patients per year), and with a margin of error set at 5% and confidence level set at 95%, a minimum sample size of 201 was required to be included

### Preoperative assessment

All patients were subjected to a thorough review of medical history, physical examination, and routine preoperative investigations, including complete blood count, serum CRP, and PCT. Pelvic-abdominal ultrasonography, magnetic resonance imaging, and pelvic-abdominal computed tomography (CT) with double contrast were performed with colonoscopy in the elective cases. Previously published ERAS protocols were followed in the present study [19–22].

### Data collected

For each intervention, data on the surgical approach (laparotomy or laparoscopy), underlying pathology, type of resection (right/left hemicolectomy, rectal resection, Hartman’s reversal, or closure of colostomy), and type of the anastomosis (stapled or hand-sewn, end-to-end, side-to-side, or end-to-side) were recorded. The choice between open and laparoscopic surgery was driven by the presence of contraindications for laparoscopy and, according to the patients’ desire, after surgeon counseling. Choosing hand-sewn or stapled anastomosis was decided according to resources available in our center.

### Postoperative assessment and outcomes

Patients were examined daily to assess their clinical condition in terms of the presence of pain, fever, hemodynamic status, abdominal examination, return of bowel function, wound condition, and hemoglobin level (if required).

The primary outcome of the study was AL which was defined as a disruption in the integrity of the intestinal wall at the anastomotic site that required surgical or radiological intervention. Upon clinical suspicion, the diagnosis of AL was confirmed with pelvic-abdominal CT with double contrast. Both clinical and radiological ALs, including those managed conservatively, were included in the present study. Clinical AL is defined as patients with evidence of a leak that needs active management, whether therapeutic or surgical intervention. A discovery at reoperation, feculent drainage , fecal debris from the incision, extravasation of contrast on enema, or the existence of air or fluid in the anastomotic area observed by computed tomographic (CT) scan were all considered anastomotic leaks [23]. Patients were then divided into two groups according to the presence or absence of AL. The two groups were compared concerning the following parameters: sex, age, underlying pathology, the urgency of intervention (elective or urgent), surgical approach (laparotomy or laparoscopy), type of resection, length of hospital stay, and postoperative morbidity and mortality. C-reactive protein and PCT levels were measured before surgery and on a daily basis until POD 5 or discharge. Normal serum level of PCT in adults is < 0.1 ng/ml and normal serum CRP is < 1 mg/dl. The assessment of the CRP, PCT, and WBCs trajectories in predicting AL was investigated prospectively. CRP was assessed by immunonephelometry on an automated dimension Vista analyzer [24] (Siemens, Erlangen, Germany). Procalcitonin was assessed with homogeneous phase sandwich enzyme-linked immunosorbent assay analysis (Brahms, Hennigsdorf, Germany) [25]. Patients were followed up for 30 days postoperatively to detect late AL.

### Statistical analysis

All statistical analyses were performed using Statistical Package for the Social Sciences, version 25.0 (IBM Corp., Armonk, NY, USA). Continuous variables were presented as mean and standard deviation (SD) if normally distributed or median and range if abnormally distributed. Categorical variables were presented as frequencies and percentages. All comparisons of continuous variables were performed using the Mann–Whitney *U* test. Biomarker trajectory was calculated as the average linear trend from day 0 to day 5 (5-day trajectory) and between every two consecutive days using linear regression. A logistic regression model for each biomarker trajectory and their combinations were used with AL as the outcome. The predicted values of the logistic regression models were used to examine the predictive performance of biomarkers trajectories using receiver operating characteristic (ROC) analysis. The respective areas under the curve (AUC) and its 95% confidence interval were calculated to evaluate the predictive performance of CRP, PCT, and WBC for AL. The sensitivity (SN), specificity (SP), negative predictive value (NPV), and positive predictive value (PPV) of these parameters were calculated using ROC analysis for continuous variables or 2 × 2 tables for binary variables (e.g., a trajectory of more than 50 mg/L increase in CRP, 0.5 ng/mL increase for PCT, or 1000/mm^3^ for WBC). *P* values of less than 0.05 were used to denote statistical significance at a 95% level of confidence. Intra-individual variability was further evaluated with three indices:

1. Within-individual standard deviation (SD)
2. Coefficient of variation (CV) which was calculated as the ratio of SD to the mean)
3. Variability independent of the mean (VIM), which was calculated as the SD divided by the within-individual mean to the power x and multiplied by the mean value of the laboratory parameter in the cohort to the power x

The power x was obtained by fitting a curve through a plot of SD against mean laboratory parameter, using the model SD=a*mean x, where x is derived by nonlinear regression [26, 27].

## Results

### Patients’ characteristics

**Fig. 1:**
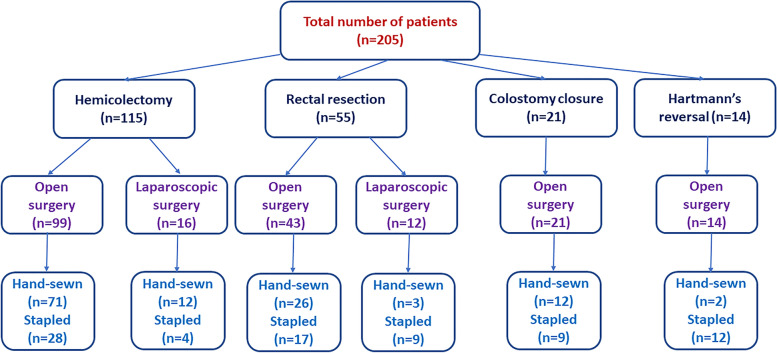
Flow chart depicting the indications for and types of colorectal anastomoses performed in the study

Overall, 217 patients who had colorectal surgery to diagnose new or previous colorectal cancer were initially screened. Twelve patients did not meet the study inclusion criteria and were excluded, and thus 205 patients were ultimately included. Males represented 56.1% of patients, while 43.9% were females. Patient age ranged from 25 to 78 years, with a mean of 56.4 years. The mean body mass index was 29.6 ± 2.9 kg/m^2^. Approximately half of the patients had chronic medical conditions, particularly diabetes mellitus (20.6%) and cardiovascular conditions (14.2%), but none had liver cirrhosis or other pathology. Approximately 84% of the patients had locally advanced tumor stages (T3/T4). A hemicolectomy was performed on 56.1% of the patients, whereas rectal resection was performed on 26.8%. Other procedures included colostomy closure (10.2%) and Hartman’s reversal (6.8%). Open surgery was performed in 86.3% of the patients and laparoscopic procedures in 13.7%. Side-to-end and end-to-end anastomoses were the most common anastomoses performed (42% and 29.3%, respectively). The hand-sewn anastomosis was performed on 6.5%, while 38.5% were stapled. The mean operation time was 166 minutes (Fig. 1, Table 1).

**Table 1:** Characteristics of the patients of the study (*n* = 205)

Variables	Frequency (%)
**Sex**	
Male	115 (56.1%)
Female	90 (43.9%)
**Age** (*years*), mean (SD), range	56.4 (13.1), 25–78
**Comorbidities**	
None	101 (49.5%)
Diabetes mellitus	42 (20.6%)
Cardiovascular	29 (14.2%)
COPD	22 (10.8%)
Renal failure	10 (4.9%)
**Tumor stage**	
T1	3 (1.5%)
T2	29 (14.1%)
T3	110 (53.7%)
T4	63 (30.7%)
**Type of operation**	
Right hemicolectomy	61 (29.8%)
Left hemicolectomy	54 (26.3%)
Rectal resection	55 (26.8%)
Closure of colostomy	21 (10.2%)
Hartman's reversal	14 (6.8%)
**Surgical approach**	
Open	177 (86.3%)
Laparoscopic	28 (13.7%)
**Type of anastomosis**	
End-to-end	60 (29.3%)
End-to-side	26 (12.7%)
Side-to-end	86 (42.0%)
Side-to-side	33 (16.1%)
**Anastomotic technique**	
Handsewn	126 (61.5%)
Stapled	79 (38.5%)
**Operation time** (*min*), mean (SD), range	166.2 (19.5), 120–200
**Hospital Stay** (*days*), mean (SD), range	10.7 (3.8), 4–21
**Postoperative complications**	
No complications	168 (81.9%)
Total complications:	37 (18.1%)
Anastomotic leakage	22 (10.7%)
Wound Infection	7 (3.4%)
Respiratory Infection	4 (1.96%)
Urinary tract infection	3 (1.46%)
Mortality	6(2.9%)
**Management of anastomotic leakage** (*n* = 22)	
Reoperation	17 (77.3%)
PCT drainage and antibiotics	5 (22.7%)

*COPD* chronic obstructive pulmonary disease, *PCT* percutaneous drainage

### Outcomes

One hundred and seventy-four patients (84.9%) were discharged without complications after a mean hospital stay of 10.7 ± 3.8 days. In contrast, thirty-one patients (15.1%) experienced complications. Wound infection was recorded in seven patients (3.4%), whereas respiratory and urinary tract infections were recorded in 1.96% and 1.46% of the patients, respectively. Anastomotic leak was detected in 22 (10.7%) patients between the 6th and 14th postoperative day (POD); 14 had clinical AL manifested by fever, pain, tachycardia, and peritonitis, and 8 had radiologically evident (subclinical) AL. Anastomotic leak was surgically treated in 77.3% of patients and was treated with percutaneous drainage and antibiotics in 22.7% (Table 1). The anastomotic leak occurred mainly after left hemicolectomy, rectal resection, and Hartman’s reversal (16.7%, 16.7%, and 14.3%, respectively). Six (2.9%) patients died after a mean of 16 PODs, 4 of whom had a rectal resection, and 2 had left hemicolectomy, but none of the deaths was related to AL.

### CRP, PCT, and WBC count measurements

The mean preoperative CRP, PCT, and WBC levels of patients who developed AL were comparable to those without AL. The mean postoperative CRP, PCT, and WBC levels in patients with AL were significantly higher than in patients without AL starting from POD2 of the 5-day observation period following surgery (Table 2).

**Table 2:** Distribution of CRP, PCT, and WBC among patients with and without anastomotic leakage at different time points (*n* = 205)

Measurements	Without leakage	With leakage	***P*** value
**CRP (mg/L)**			
Preoperative	3.8 (1.8)	3.69 (2.09)	0.701
POD 1	19.80 (8.25)	19.91 (8.16)	0.891
POD 2	44.96 (28.45)	66.03 (24.77)	0.002^*^
POD 3	56.40 (45.02)	111.13 (51.00)	< 0.001^*^
POD 4	61.02 (56.90)	142.14 (81.60)	< 0.001^*^
POD 5	58.61 (65.57)	150.90 (120.17)	0.006^*^
**PCT (ng/mL)**			
Preoperative	0.57 (0.34)	0.61 (0.30)	0.478
POD 1	0.84 (0.45)	1.28 (0.55)	< 0.001^*^
POD 2	1.32 (0.58)	2.22 (1.15)	< 0.001^*^
POD 3	1.68 (0.92)	3.39 (1.49)	< 0.001^*^
POD 4	1.99 (0.95)	4.75 (2.21)	< 0.001^*^
POD 5	2.21 (1.08)	6.31 (2.65)	< 0.001^*^
**WBC (**× **1000/mm**^**3**^**)**			
Preoperative	6.41 (1.60)	5.85 (0.88)	0.187
POD 1	6.96 (1.44)	6.98 (0.84)	0.983
POD 3	7.99 (1.57)	8.79 (1.13)	0.014^*^
POD 5	8.34 (3.34)	10.01 (1.51)	< 0.001^*^

*CRP* C-reactive protein, *PCT* procalcitonin, *WBC* white blood cell count, *POD* postoperative day

^*^Statistically significant difference at *p* values of less than 0.05

Values are presented as mean (standard deviation)

The predictive power of CRP, PCT, and WBC levels on individual time points from POD 1 to POD 5 showed that the AUC of PCT was higher than that of CRP and WBC at each corresponding time point, particularly on PODs 3, 4, and 5 (AUC = 0.84, 0.85, and 0.89, respectively).

On other PODs, the AUC for PCT ranged from 0.73 to 0.89, whereas those of CRP and WBC ranged from 0.51 to 0.68 and from 0.51 to 0.89, respectively. On POD 5, a cutoff value of 4.93 ng/mL for PCT had the highest SN, SP, and NPV (77.3%, 96.7%, and 97.3%, respectively). However, the cutoff values for CRP and WBC had their highest SN on PODs 1–3 and their highest SP on PODs 3–5. The highest NPV was achieved for CRP ≥ 114.1 mg/L and WBC ≥ 9.02 × 1000/mm^3^ on PODs 3 and 5, respectively (97.5% and 97.7%, respectively) (Table 3).

**Table 3:** Predictive performance of CRP, PCT, and WBC for anastomotic leakage on isolated time points

Isolated time points	AUC	95% CI	Cutoff values	SN	SP	PPV	NPV
**CRP (mg/L)**							
POD 1	0.51	0.44–0.58	12.5	90.9	24.0	12.6	95.7
POD 2	0.70^*^	0.63–0.76	62.6	81.8	67.2	23.1	96.9
POD 3	0.76^*^	0.69–0.81	114.1	81.1	85.2	40.0	**97.5**
POD 4	0.75^*^	0.68–0.81	168.4	72.7	94.5	61.5	96.6
POD 5	0.68^*^	0.61–0.74	210.5	59.1	97.8	76.5	95.2
**PCT (ng/mL)**							
POD 1	0.73^*^	0.66–0.79	1.30	50.0	88.0	33.3	93.6
POD 2	0.73^*^	0.67–0.79	1.89	63.6	86.3	35.9	95.2
POD 3	0.84^*^	0.79–0.89	2.60	77.3	90.7	50.0	**97.1**
POD 4	0.85^*^	0.80–0.90	3.65	72.7	94.5	61.5	96.6
POD 5	0.89^*^	0.84–0.93	4.93	77.3	96.7	73.9	97.3
**WBC (**× **1000/mm**^**3**^**)**							
POD 1	0.50	0.45–0.59	6.11	90.9	30.6	13.6	96.6
POD 3	0.66^*^	0.59–0.73	7.50	91.0	41.5	15.7	97.4
POD 5	0.79^*^	0.73–0.85	9.02	86.4	71.0	26.4	97.7

*CRP* C-reactive protein, *PCT* procalcitonin, *WBC* white blood cell count, *SN* sensitivity, *SP* specificity, *PPV* positive predictive value, *NPV* negative predictive value, *POD* postoperative day

^*^Statistically significant different AUC from the reference diagonal line at *p* values of less than 0.05

**Fig. 2:**
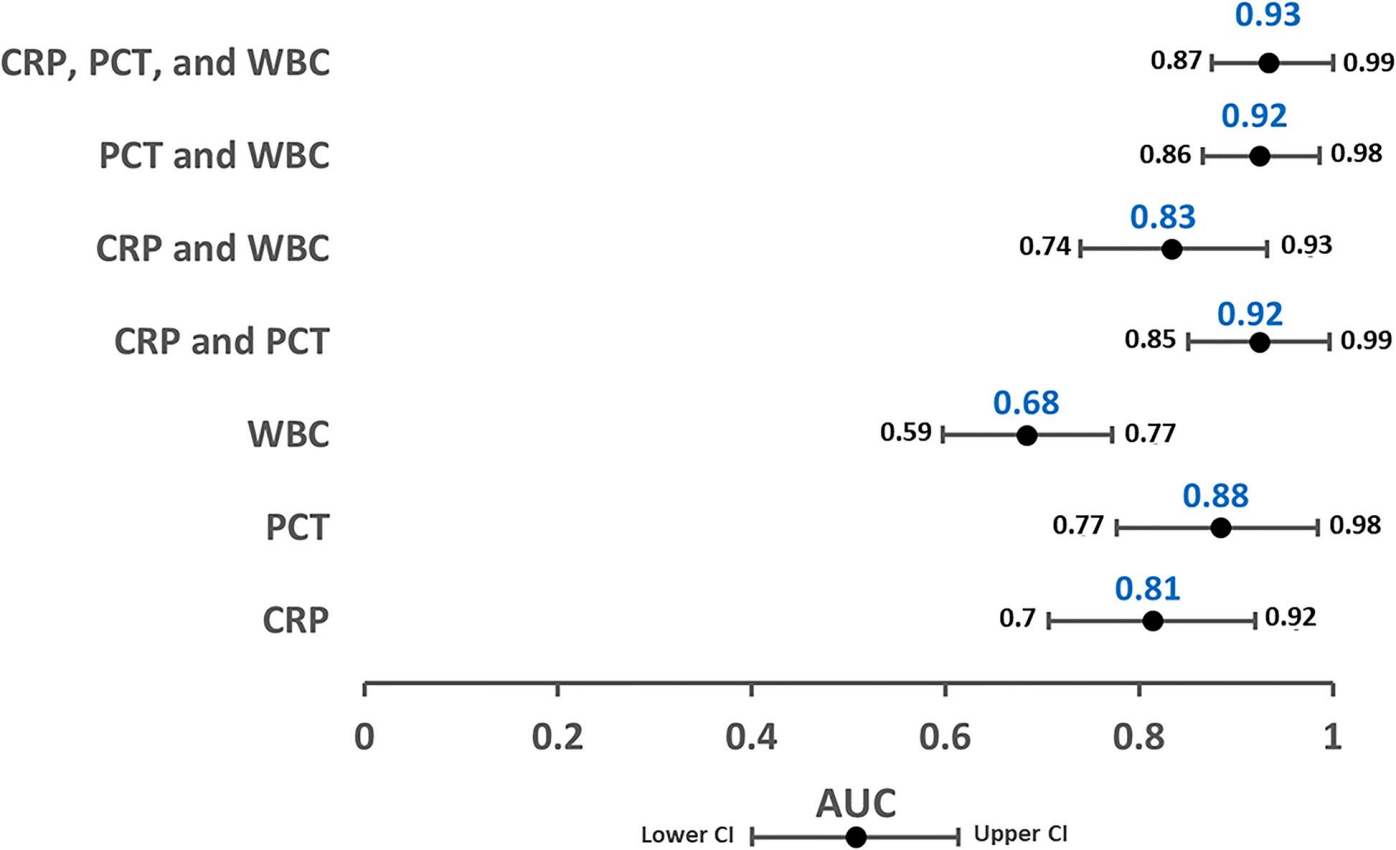
Area under the receiver operator curve estimates for the models predicting anastomotic leakage, including CRP, PCT, and WBC trajectories and their combinations, over the 5-day postoperative observation period. CRP, C-reactive protein; PCT, procalcitonin; WBC, white blood cell count; AUC, area under the curve

Trajectory analysis of PCT, CRP, and WBC count and their combinations over the 5-day observation period revealed that the PCT trajectory had the highest AUC compared with CRP and WBC (0.88 vs. 0.81 and 0.68, respectively). The predictive assessment of the PCT trajectory showed a substantial improvement when combined with the trajectories of either CRP or WBC, or both (AUC: 0.92, 0.92, or 0.93, respectively) (Fig. 2). Follow-up of the patients revealed no AL beyond 30-days after surgery.

Furthermore, an increase of CRP of more than 50 mg/L between any two consecutive days had an AUC of 0.84 (SN: 90.9%, SP: 77.6%, PPV: 32.7%, NPV: 98.6%) with the highest predictive performance between POD 2 and 3 (AUC: 0.85, SN: 81.8%, SP: 88.5%, PPV: 46.1, NPV: 97.6%). Likewise, an increase of PCT more than 0.5 ng/ml between any two consecutive days had an AUC of 0.93 (SN: 95.5%, SP: 89.6%, PPV: 52.4%, NPV: 99.4%) with the highest predictive value between POD 4 and 5 (AUC: 0.92, SN: 86.4%, SP: 97.3%, PPV: 79.1, NPV: 98.3%). In contrast, WBC trajectory had less value in predicting AL. An increase in WBC of more than 1000/mm^3^ between any two consecutive days had an AUC of 0.75 (SN: 100%, SP: 49.2%, PPV: 19.1, NPV: 100%) with the highest predictive value between POD 3 and 5 (AUC: 0.84, SN: 90.9%, SP: 77.0%, PPV: 32.2, NPV: 98.6%) (Table 4).

**Table 4:** Predictive performance of CRP, PCT, and WBC Trajectories for the anastomotic leak

Trajectories	AUC	95% CI	SN	SP	PPV	NPV
**CRP > 50 mg/l**						
**Between any 2 days**	0.84^*^	**0.79–0.89**	**90.9**	**77.6**	**32.7**	**98.6**
From POD 1 to POD 2	0.83^*^	0.76–0.88	86.4	80.3	34.5	98.0
From POD 2 to POD 3	0.85^*^	0.80–0.90	81.8	88.5	46.1	97.6
From POD 3 to POD 4	0.81^*^	0.75–0.86	72.7	89.6	45.6	96.5
From POD 4 to POD 5	0.78^*^	0.71–0.83	59.1	96.2	64.9	95.2
**PCT > 0.5 ng/ml**						
**Between any 2 days**	0.93^*^	**0.88–0.96**	**95.5**	**89.6**	**52.4**	**99.4**
From POD 1 to POD 2	0.79^*^	0.73–0.84	68.2	89.6	44.0	95.9
From POD 2 to POD 3	0.86^*^	0.80–0.90	77.3	94.5	62.9	97.2
From POD 3 to POD 4	0.87^*^	0.81–0.91	77.3	96.2	70.8	97.2
From POD 4 to POD 5	0.92^*^	0.87–0.95	86.4	97.3	79.1	98.3
**WBC > 1.0 (**× **1000/mm**^**3**^**)**						
**Between any 2 days**	0.75^*^	**0.68–0.80**	**100**	**49.2**	**19.1**	**100**
From POD 1 to POD 3	0.72^*^	0.65–0.78	86.4	57.4	19.5	97.2
From POD 3 to POD 5	0.84^*^	0.78–0.89	90.9	77.0	32.2	98.6

*CRP* C-reactive protein, *PCT* procalcitonin, *WBC* white blood cell count, *SN* sensitivity, *SP* specificity, *PPV* positive predictive value, *NPV* negative predictive value, *POD* post-operative day

^*^Statistically significant different AUC from the reference diagonal line at *p* value < 0.05

The SD and CV indices of intra-individual variability showed that intra-individual variability of CRP, PCT and WBC was significantly different between patients with AL, and those without AL; patients with AL showed higher variability. Although VIM of CRP in AL was significantly higher than non-AL, the difference for PCT or WBC was not significant (Table 5).

**Table 5:** Day-to-day intra-individual variability Indices of CRP, PCT, and WBC for anastomotic leakage

Within table-individual variability		No AL	AL	***Mean difference (95% CI)***	***p*** value
**CRP**	SD	30.72 (24.9)	74.67 (33.8)	43.9 (32.4–55.5)	< 0.001*
	CV	0.68 (0.22)	0.93 (0.14)	0.25 (0.16–0.35)	< 0.001*
	VIM	31.90 (9.78)	41.45 (8.25)	9.56 (5.27–13.8)	< 0.001*
**PCT**	SD	0.66 (0.45)	2.27 (1.09)	1.61 (1.12–2.10)	< 0.001*
	CV	0.47 (0.14)	0.71 (0.21)	0.24 (0.14–0.33)	< 0.001*
	VIM	0.87 (0.37)	0.85 (0.27)	− 0.02 (− 0.18–0.15)	0.673
**WBC**	SD	1.25 (1.55)	1.92 (0.60)	0.67 (0.01–1.33)	< 0.001*
	CV	0.17 (0.13)	0.24 (0.06)	0.08 (0.02–0.13)	< 0.001*
	VIM	1.67 (2.19)	1.63 (0.59)	− 0.03 (− 0.96–0.89)	0.062

*OR* odds ratio, *CI* confidence interval, *SD* standard deviation, *CV* coefficient of variation, *VIM* variability independent of the mean

*Statistically significant *p* value (< 0.05); Mann-Whitney test

## Discussion

Anastomotic leak is considered a common and serious complication of colorectal surgery. Early recognition of AL is imperative to reduce mortality and morbidity. However, early detection of AL can be challenging because early clinical and radiologic signs are rather non-specific. Therefore, accurate biomarkers for early AL detection are highly required [28, 29]. This study found a strong association between AL and the trajectory of CRP, PCT, and WBC over 5 PODs. The area under the curve (AUROC) is a commonly used metric to determine the discriminatory power of a diagnostic method. The AUROC has the highest value of 1.0, signifying a (theoretically) flawless test. AUROC of 0.5 implies no discriminative value and is depicted as a straight, diagonal line running from the bottom left corner to the top right corner. Any measure with an AUROC value > 0.9 has high preferential strength [30]. The solo trajectory of PCT was the most reliable biomarker compared to CRP and WBC for detecting AL, and its combination with the CRP and WBC trajectories provided the maximum AL diagnostic accuracy with an AUC of 0.93.

Being a serious complication [31], there were several attempts to investigate the risk factors for developing AL [32]. Paliogiannis et al., as repoted the neutrophil to lymphocyte (NLR), derived neutrophil to lymphocyte (dNLR), lymphocyte to monocyte (LMR), and platelet to lymphocyte (PLR) ratios, however, the AUC as not exceed 0.744; 95% CI 0.719–0.768 in predicting AL [33].

Recently, PCT has been studied as a marker of early inflammatory changes earlier than CRP. PCT tends to reflect the magnitude of the systemic inflammatory response in the first 12 h postoperatively, particularly in bacterial infection with a systemic response facilitating early diagnosis of AL, allowing for early therapeutic interventions, and conferring better outcomes [34]. Nonetheless, PCT remains a questionable biomarker for AL. Smith et al. reported lower accuracy of the PCT trajectory than CRP (AUC: 0.763 vs. 0.961, respectively) [12]. In contrast, Garcia-Granero et al. found PCT to be the most accurate biomarker with an AUC of 0.86 [35], which is in line with the findings of our study.

Several studies have emphasized the utility of CRP as a diagnostic indicator for AL. CRP has an adequate discriminatory capacity for AL with an AUC varying from 0.69 to 0.87 [36–39]. The PREDICT study assessed the trajectory of CRP levels along with the PODs on 833 patients. The study concluded that the CRP trajectory could accurately exclude postoperative AL [40]. However, the PREDICT study did not assess the accuracy of combined trajectories of other potential biomarkers. Our study found biomarker trajectories to be more predictive of AL than their individual values at each POD. Moreover, the combination of various biomarker trajectories can maximize the predictive power.

Previous studies have demonstrated the possibility of using CRP as a marker for infection-related complications after gastrointestinal operations [41]. It has been shown that the serum concentration of CRP significantly increases immediately after surgery and returns to normal on POD 3 in patients without complications. According to Yeung et al., the increase in CRP after rectal resection suggests AL, and its evaluation in the postoperative period can be useful for early detection of AL. With a cutoff value of 148 mg/dL on POD 3, the SN and SP of CRP was 95% [42]. In another study, Ortega-Deballon et al. reported that CRP is a good predictor for AL, with an AUC of 0.8 on POD 4 [37].

Similarly, in a study by Zawadzki et al., serum CRP was markedly elevated at the third POD among patients with AL [28]. Messias et al. [43] did not find any statistically significant alterations in serum CRP levels in the first 3 PODs. However, starting from POD 4, serum CRP values in patients with AL were significantly higher than those in patients without AL The highest CRP levels were observed in patients with AL on POD 5. Su’a et al. [44] analyzed 11 studies on AL and found a broad range of CRP cutoff values on the third and fourth PODs, varying from 94 to 190 mg/L. Medications, such as corticosteroids and statins may change this reaction, reduce serum CRP thresholds, and alter the perception of cutoff values by 22%. Singh et al. [36] conducted a systematic review of seven studies, including more than 2400 patients [41], and found that CRP levels were comparable on PODs 3, 4, and 5.

Smith et al. reported CRP as the most accurate biomarker for the anastomotic leak. However, the consistency of the CRP trajectory in the present study was not observed [12]. Although the AUC values did not exceed those known to be highly informative (0.81), CRP monitoring was proper as indicated by its NPV (97.5%) along with its high SP starting at POD 3. These findings were supported by the PREDICT study [40], which has reported an AUC of 0.85 and NPV of 0.95 for CRP.

According to our study, the PCT trajectory is the best solo predictor of major AL at PODs 1, 3, and 5 with a maximum AUC of 0.89 on POD 5 with a cutoff value of 4.93 mg/L and an NPV of 97.3%. Moreover, it can give a maximum discriminatory value for AL diagnosis when combined with the 5-day trajectories of CRP and WBC. In terms of WBC as AL marker, it was not a point of investigation by some researchers [45, 46] or it has been reported that there was no relation to AL, however, they have included only symptomatic AL patients and isolated values rather than trajectory [47, 48]. PCT has also been investigated recently as an early marker for septic complications after surgery. The iCral study [49] and Spoto et al. [50] have shown that high postoperative PCT levels are associated with significant complications and suggested that patients with high postoperative PCT levels should undergo imaging studies to search for surgical complications. A meta-analysis by Cousin et al. confirmed that PCT, measured on POD 5, is a helpful biomarker for the early diagnosis of intra-abdominal infection, including AL, after colorectal surgery [51]. In another study, Giaccaglia et al. showed that low levels of these two biomarkers on PODs 3 and 5 were associated with a low risk of AL [52].

The strengths of the present study include the assessment of the predictive power of three different biomarkers separately and combined, which was not reported on previously. Limitations of the study include the single-center nature and relatively small numbers of patients included. The predictive utility of the combined trajectory of the biomarkers assessed in the current study needs to be investigated in more extensive multicenter studies. Furthermore, data on other confounding variables such as diabetes, immunosuppressive medical conditions, smoking were unavailable. In addition to AL, many other infectious diseases (for example: postoperative pneumonia) cause PCT, CRP and WBC to rise. Thus, we have considered using “propensity score matching”; however, the sample size of our study, particularly the number of patients with AL, was too small to perform this type of analysis which may render it underpowered. Therefore, we preferred not to lose the power of the study and proceed with the current approach of data analysis.

## Conclusion

This study provides evidence on the usefulness of the combined triad of PCT, CRP, and WBC trajectories as accurate biomarkers for AL after colorectal surgery, particularly on PODs 3 and 5. Furthermore, this combination can be a reliable predictor for early patient discharge, which would be highly beneficial to ERAS programs.

## Data Availability

All data are available on reasonable request
