# Correction to: Role of the triad of procalcitonin, C-reactive protein, and white blood cell count in the prediction of anastomotic leak following colorectal resections

**DOI:** 10.1186/s12957-022-02540-2

**Published:** 2022-03-01

**Authors:** Haidi Abd El Zaher, Waleed M. Ghareeb, Ahmed M. Fouad, Khaled Madbouly, Hamada Fathy, Tomas Vedin, Marcus Edelhamre, Sameh H. Emile, Mohammed Faisal

**Affiliations:** 1 Surgical Oncology Unit, Department of Surgery, Faculty of Medicine, Suez Canal University Hospital, Ismailia, Egypt; 2 Gastrointestinal Surgery Unit, Department of Surgery, Faculty of Medicine, Suez Canal University Hospital, Ismailia, Egypt; 3 Faculty of Medicine, Suez Canal University, Ismailia, Egypt; 4 Department of Public Health, Community Medicine, Occupational & Environmental Medicine, Faculty of Medicine, Suez Canal University, Ismailia, Egypt; 5 Colorectal Surgery Unit, Alexandria University, School of Medicine, Alexandria, Egypt; 6 Department of Surgery, Helsingborg Hospital, University of Lund, 251 87 Helsingborg, Sweden; 7 Colorectal Surgery Unit, General Surgery Department, Mansoura University Hospital, Mansoura, Egypt; 8 General Surgery Department, Sahlgrenska University Hospital, Gothenburg, Sweden

**Correction to: World J Surg Oncol 20, 33 (2022)**

**https://doi.org/10.1186/s12957-022-02506-4**

Following the publication of the original article [1], the affiliation of the 3rd author named Ahmed M. Fouad was written wrongly as

4 Department of Public Health, Community Medicine, Occupational & Environmental Medicine, Faculty of Medicine, Suez Canal University, Fuzhou, China,

It should be

4 Department of Public Health, Community Medicine, Occupational & Environmental Medicine, Faculty of Medicine, Suez Canal University, Ismailia, Egypt .

In the Authors contribution section, „HA, HF, AF, and WMG carried out the study and analyzed the data“ should be „HA, HF, AF, MF and WMG carried out the study and analyzed the data“

The original article has been updated.

## References

[1] El Zaher HA, Ghareeb WM, Fouad AM (2022). Role of the triad of procalcitonin, C-reactive protein, and white blood cell count in the prediction of anastomotic leak following colorectal resections. World J Surg Onc.

